# The Need for Female Factory Inspectors

**Published:** 1891-10-10

**Authors:** 


### The Need for Female Factory Inspectors.

There are, no doubt, a certain number of women who eagerly advocate the entrance of their sex into any and every occupation in life, not because it is suited to their capacities, but because they are consumed with a burning desire to try their strength against that terrible tyrant, man. With such women we have little or no sympathy, for it is abundantly evident that, as Tennyson says, "Woman is not undevelopt man, But diverse"; and nothing but harm can come of her trying to prove that her sphere and man's are identical. Nevertheless, it may well happen that now and again we find ourselves in agreement with these militant fair ones as to the advisability of certain employments being thrown open to women. Only, we urge that this should be done, not because we are anxious that women should have a finger in every pie, but because we believe that such employments are really suitable for them, and not only so, but that certain departments of them can be far more efficiently attended to by women than by men.

Among such employments must be classed, we feel sure, the inspection of factories and workshops where women are employed. A male inspector is no doubt very useful in such places, but it seems to us self-evident that a female inspector would be far more so. This for several reasons. For one thing, there are, as has been well said, "many questions concerning the cleanliness and decency of the surroundings of female labour which a natural delicacy would prevent a man from interfering with"—at any rate, except in the most general way; and it is hardly to be expected, or even wished, that the workwomen themselves will call his attention to such sanitary defects as may exist, which they might do with the greatest ease if the inspector was a woman.

Again, it is far easier for a woman than for a man to ascertain whether the rules as to the length of the working-day are being broken or not. Hear Miss Clementina Black's testimony on this point. "It is difficult to see what a male inspector can do, who, arriving at eleven o'clock at night in a dressmaking work-room, finds all the workers vanished, and is informed that the outdoor hands are gone home, and the indoor hands asleep in their bed-rooms. He has the fullest conviction that all the workers, indoor and outdoor, each with her thimble still on her finger, have been hustled into those bed-rooms, but he cannot go and see. . . . This has happened when I myself have given information to the inspector that the girls would be kept late, and when I have known afterwards that they were so kept." There is a good deal of prating nowadays about an eight-hours' day; would it not be as well if we devoted our attention first of all to seeing that a *twelve*-hours' day is not exceeded?

Sometimes, indeed, the poor workers actually need protection from themselves, favouring the evasion of the law, and hailing with joy the prospect of an hour or two's addition to their labours—a sad proof, we fear, of the meagreness of their earnings. Others feel it a bitter blow, but have to put up with it for fear of losing their places. We quote the testimony of a young milliner, who wrote to the *St. James's Gazette* some little time ago on the subject; her letter bears every mark of being a simple and truthful statement of facts. "Perhaps you would not believe it, but it is true all the same, that in many a house of business the girls are kept working till half-past ten and eleven o'clock day after day through the best part of the season. That may not seem very much, I daresay, when you hear it told, but it is bad enough when you have to do it. Where I am, there are twenty girls all sitting round three oblong tables in a room not bigger than an ordinary drawing room, with only one window. You can think what the air of that room is like in July, when we have all been working in it for twelve hours. . . Then, when eight o'clock comes, and you are dying to get out and have a mouthful of fresh air, you are told that you will have to stop for an hour longer, or perhaps two or three hours. I have seen some of the younger girls put their heads down and cry quietly when they heard they could not go, and I have felt like crying myself if it was any use."

Once more, will it be disputed that a woman is less likely to be deceived than is a man by the plausible statements of a female employer or forewoman? There is a natural gallantry about a man which makes him very reluctant to seem to question the statements of a member of the fair sex; and, moreover, we have to reckon with that sort of glamour which many a woman knows how to throw over a man, be he ever so sensible, ever so business-like. "Men were deceivers ever," says the old song; but we fancy that even those who are all for male inspectors only, will admit, perhaps even from their own bitter experience, that it is not only men who are adepts in this art. As the little milliner quoted above innocently puts it: "Madame Célimène is a very nice lady, with very nice manners, and when she says to the inspector that it is all right, of course he does not doubt her." Be it understood that we have no wish to say or to insinuate a word against the male inspector, who is, we are sure, a most sensible and intelligent personage; still, human nature is human nature, and we think the proverb, "Set a thief to catch a thief," should have as a companion proverb, "Set a man to catch a man, and a woman to catch a woman."

But have we any evidence to show that a woman can satisfactorily fulfil these duties? Well, we have this much, that in New York some half-dozen lady inspectors, paid at the same rate as men, have actually been appointed, and have been at work for a year; and that in our own country Miss Margaret E. Scott has gone through the entire course of training required of our sanitary inspectors, and passed the examination established by the Council of the Sanitary Institute. If a woman can qualify herself for a sanitary inspector, most certainly she can qualify herself for a factory inspector; and we much hope that she will shortly be encouraged to try.

The Home Secretary has spoken of the "great waste of power" which would result from the duplication of inspectors in every district, and the "difficulty of arranging the work." But where there is a will, there is a way; and it might surely be arranged, say, by enlarging the districts, or by giving a woman inspector charge of the women's workshops in two districts instead of one, so that there should be very little "waste of power." As Sir John Gorst (a Tory, too!) has put it: "If we are embarking on a course of social legislation, we shall have to avail ourselves in the future, far more than we have done as yet, of the counsel and experience of women. There is much which a woman can see and understand and deal with, which is a closed book to men."

---

There have been many unsuitable and cumbersome mediums of exchange in the world, some of which still exist in China. In the large towns of that country, silver is still used in bulk by weight; but owing to the inconvenience of dividing it, there is a drawback to its use in the country. The ingenuity of the wily Celestial has been exercised with a view to overcoming the difficulty in a somewhat novel manner. As the credit of the native bankers is not widely enough established to give an extended currency to their notes, the Celestial, in parts of the Hankow districts, has resorted to the use of opium. It is found to have the following advantages in facilitating the exchange of commodities; it is in great demand, is light and portable, and is easily divided into small quantities.

